# The Efficacy of Nerve Growth Factor Antibody for the Treatment of Osteoarthritis Pain and Chronic Low-Back Pain: A Meta-Analysis

**DOI:** 10.3389/fphar.2020.00817

**Published:** 2020-06-30

**Authors:** Si Yang, Yu Huang, Ziqi Ye, Lu Li, Yu Zhang

**Affiliations:** ^1^ Department of Pharmacy, The First Affiliated Hospital, College of Medicine, Zhejiang University, Hangzhou, China; ^2^ Department of Laboratory Medicine, Hubei NO.3 People’s Hospital of Jianghan University, Wuhan, China

**Keywords:** anti-NGF antibody, osteoarthritis pain, chronic low-back pain, immunological treatment, meta-analysis

## Abstract

**Background:**

Nerve growth factor (NGF) plays a crucial role in pain modulation and is being considered as a new therapeutic target for pain therapy. The purpose of this meta-analysis was to study the efficacy of anti-NGF antibodies for the treatment of osteoarthritis pain and chronic low-back pain, and to provide evidence and direction for further research and practice.

**Methods:**

PubMed, Embase, Wanfang Data, and China National Knowledge Infrastructure (CNKI) were searched from inception to November 30, 2019. Eligible studies should include randomized clinical trial-based investigations of anti-NGF antibody treatment for osteoarthritis pain and chronic low-back pain. Pooled overall mean changes from baseline to check point in the Western Ontario and McMaster Universities Arthritis Index (WOMAC) measures of pain, physical function, and Patient’s Global Assessment (PGA) were calculated with either a fixed-effects model or a random-effects model, depending on the tests for heterogeneity. Sensitivity analysis and bias of publication were assessed.

**Results:**

A total of seven studies (3890 patients) were included in this meta-analysis. The pooled analysis showed a statistically significant reduction in the WOMAC pain (standardized mean difference (SMD) = -2.22, 95% confidence interval (CI) = -3.44 to -0.99, Z = -3.55, P = 0.0004; I^2^ = 99%), the WOMAC Physical Function (SMD = -2.76, 95% CI = -4.22 to -1.30, Z = -3.71, P = 0.0002; I^2^ = 99%), and the PGA Index (SMD = -2.76, 95% CI = -4.42 to -1.09, Z = -3.24, P = 0.0012; I^2^ = 99%). Pooled differences of adverse events rates in experimental and control groups was 0.11 (95% CI = 0.02 to 0.20, Z = 2.41, P = 0.016; I^2^ = 83%).

**Conclusion:**

Our meta-analysis data indicate that anti-NGF antibodies can relieve pain and improve function in patients with osteoarthritis pain and chronic low-back pain.

## Introduction

Osteoarthritis (OA) is a 00degenerative articular disease and is one of the most common di0sorders among the elderly (Zhang et al., 2019). The major symptoms include pain, stiffness, swelling, tenderness, and joint movement disfunction, which has a serious impact on patients. Pain is the most prevalent symptom of OA (Felson, 2006). Unfortunately, current treatments only play a role in pain relief and symptoms control; as for the physical functional capacity improvement, radical therapy modalities have yet to appear (Zhang et al., 2010). Statistically, over two thirds of adults have been affected by low-back pain (LBP) during their lifetime, and in some cases it may progress to a chronic state (lasts >3 months), resulting in high rates of morbidity, disability, and productivity declines (Guo et al., 1999; Gore et al., 2012). Therapeutic measures for chronic LBP are therefore aimed at pain remission (Kivitz et al., 2013). Clinical therapeutics of both OA and chronic LBP is a difficult issue (Chou and Huffman, 2007; Chou et al., 2007; Conaghan, 2018; Schmelz et al., 2019). Rehabilitation, pharmacological therapy, psychotherapy, and other modalities are recommended for many of these chronic pain conditions (Chou et al., 2009; Stanos et al., 2016). Although pharmacotherapy is an important treatment approach, many medications are proven to give rise to drug-related adverse effects and complications, as well as other undesirable consequences (Kissin, 2010; McAlindon et al., 2014). Therefore, there is an unmet need for an effective and safe resolution for chronic pain conditions.

For two decades, the growing investigation on nerve growth factor (NGF) and its relevant molecular targets has provided a completely original mode of disease therapeutics, especially for the treatment of chronic pain (Chang et al., 2016; Schmelz et al., 2019). It has been found that NGF may regulate pain through nociceptor sensitization (Loeser et al., 2012; Lin et al., 2014). Levels of NGF have been found to be upregulated among peripheral nerve injury models in several preclinical studies (Zahn et al., 2004; Wild et al., 2007; Cruz et al., 2011; Hakim et al., 2011; Zhu et al., 2012). Clinically, concentrations of NGF are elevated in patients with a chronic pain state (e.g. osteoarthritis, chronic headaches, interstitial cystitis, pancreatitis, cancerous pain, diabetic neuropathy, etc.) (Ugolini et al., 2007; Wild et al., 2007; Chang et al., 2016). In rodent models, pain-related behaviors disappeared after the use of NGF antagonists (Sevcik et al., 2005; Koewler et al., 2007; Wild et al., 2007), which provided rationale for the application of NGF antagonists to achieve significant relief of chronic pain. Furthermore, hyperalgesia and pain were reduced by the application of anti-NGF antibodies or IgG fusion proteins, both of which have the potential to inhibit NGF activities (Lane et al., 2010; Schnitzer et al., 2011; Kivitz et al., 2013). Recent clinical studies presented promising outcomes for anti-NGF drugs in the treatment of OA and chronic LBP in human beings (Nagashima et al., 2010; Katz et al., 2011; Brown et al., 2012; Brown et al., 2013; Kivitz et al., 2013; Sanga et al., 2013; Spierings et al., 2013; Balanescu et al., 2014; Ekman et al., 2014; Tiseo et al., 2014; Gow et al., 2015; Schnitzer et al., 2015; Mayorga et al., 2016; Sanga et al., 2016; Slatkin et al., 2019). Fasinumab, fulranumab, and tanezumab are three NGF-Abs undergoing clinical trials (Schmelz et al., 2019). Moreover, clinical trials of inflammatory pain relevant to OA and chronic LBP reported the most consistent efficacy in pain alleviation (Bannwarth and Kostine, 2017).

Based on the current clinical trials investigating anti-NGF agents, we endeavored to present more powerful evidence by synthesizing the results in a meta-analysis. The purpose of this meta-analysis was to study the efficacy of anti-NGF antibodies for the therapy of OA and chronic LBP and to provide a reference for the upcoming clinical trials associated with anti-NGF antibodies.

## Materials and Methods

### Statement

All studies included in this meta-analysis had been published and declared ethical approval, and we did not collect or utilize any raw data of these results, therefore no ethical approval was needed for this meta-analysis study. This meta-analysis was conducted on the basis of the Preferred Reporting Items for Systematic Reviews and Meta-analysis (PRISMA) (Moher et al., 2010).

### Literature Search and Study Selection

We systematically searched the databases PubMed, Embase, Wanfang Data, and China National Knowledge Infrastructure to retrieve studies from inception to November 30, 2019. Both Chinese and English language studies were considered. The following key terms were used for the database research: nerve growth factor antibody, fasinumab, fulranumab, tanezumab, osteoarthritis pain, OA, and chronic LBP. The references of the articles included were also searched in case of any additional studies not previously identified in the initial literature search.

Inclusion criteria of studies eligible for this meta-analysis were as follows: either full-texts or abstracts of randomized controlled trials (RCTs) that included patients with osteoarthritis pain and/or chronic low-back pain that evaluated the efficacy of anti-NGF agents. Outcomes included Western Ontario and McMaster Universities Arthritis Index (WOMAC) measures of pain, physical function, Patient’s Global Assessment (PGA) (Cohen and Lee, 2015), and rates of adverse events. Studies had to report at least one indicator assessed at the end of the intervention period or at a follow-up point after randomization. If studies recruited participants over the same period or in the same study centers, only the study with the maximum sample size or yielding the most pertinent outcomes was included to avert duplications. Exclusion criteria included case reports, review articles, news, conference abstracts with unavailable indicators, and editorials.

Two reviewers, Ziqi Ye and Yu Zhang, independently screened the titles of studies and checked the full-texts or abstracts for eligibility confirmation. When disagreement occurred, they discussed their arguments, and a third reviewer, Yu Huang, was involved in the case that no consensus was achieved.

### Data Extraction and Quality Assessments

Two reviewers, Si Yang and Lu Li, independently extracted data from the eligible studies including: study name, the studied pain condition, the sample size, the mean age of participants, the percentage of included women, content of the experimental and control intervention, and the indicators used in the study. Disagreements were checked by a third investigator, Yu Huang, until consensus was finally reached. As previously mentioned, mean alterations from baseline to check point in the WOMAC measures of pain, physical function, PGA Index, and difference of adverse events rates were indicators to be pooled.

The risk of bias in individual studies were assessed in seven domains: sequence generation, allocation concealment, blinding of participants and personnel, blinding of outcome assessment, incomplete outcome data, selective outcome reporting, and other sources of bias. Of note, other bias refers to “important concerns about bias not covered in the other domains in the tool” (Higgins et al., 2011), they mainly include bias caused by early termination of a trial due to early benefit or to patients who were enrolled *via* unvalidated outcome measures or diagnostic criteria. They were evaluated by reviewers’ empirical judgment according to the prescribed protocol of this study.

### Statistical Analysis

We used the R 3.6.1 software and Review Manager 5.3 Software for statistical analyses. The standardized mean differences (SMD) or rate difference (RD) of outcomes, along with respective 95% confidence intervals (CIs), were calculated for each analysis. A Cochran Q test was used for heterogeneity evaluation between studies and an I² statistic was used to investigate the magnitude of the heterogeneity. The magnitude of heterogeneity was classified by the I² with: I² > 25%, I² > 50%, and I² > 75% representing moderate, substantial, and considerable heterogeneity, respectively (Higgins et al., 2003; Cumpston et al., 2019). We used a random-effects model or a fixed-effects model to calculate the pooled effects and their respective 95% CIs. The methods depended on: if I² value was >50%, a random-effects model was used, otherwise a fixed-effects model was used. Sensitivity analysis was conducted in order to assess the stability of pooled outcomes. We used a Rosenberg’s Fail-safe N approach to assess potential publication bias (Rosenberg, 2005). A fail-safe number is defined as the number of studies with non-significance or that were unpublished that would be needed to be enrolled in a meta-analysis to turn a statistically significant result into non-significant one (Rosenberg, 2005; Muller et al., 2019). Funnel plots were constructed to visualize possible asymmetry. A p value less than 0.05 was considered to be of statistical significance.

## Results

### Study Selection and Characteristics

**Figure 1:**
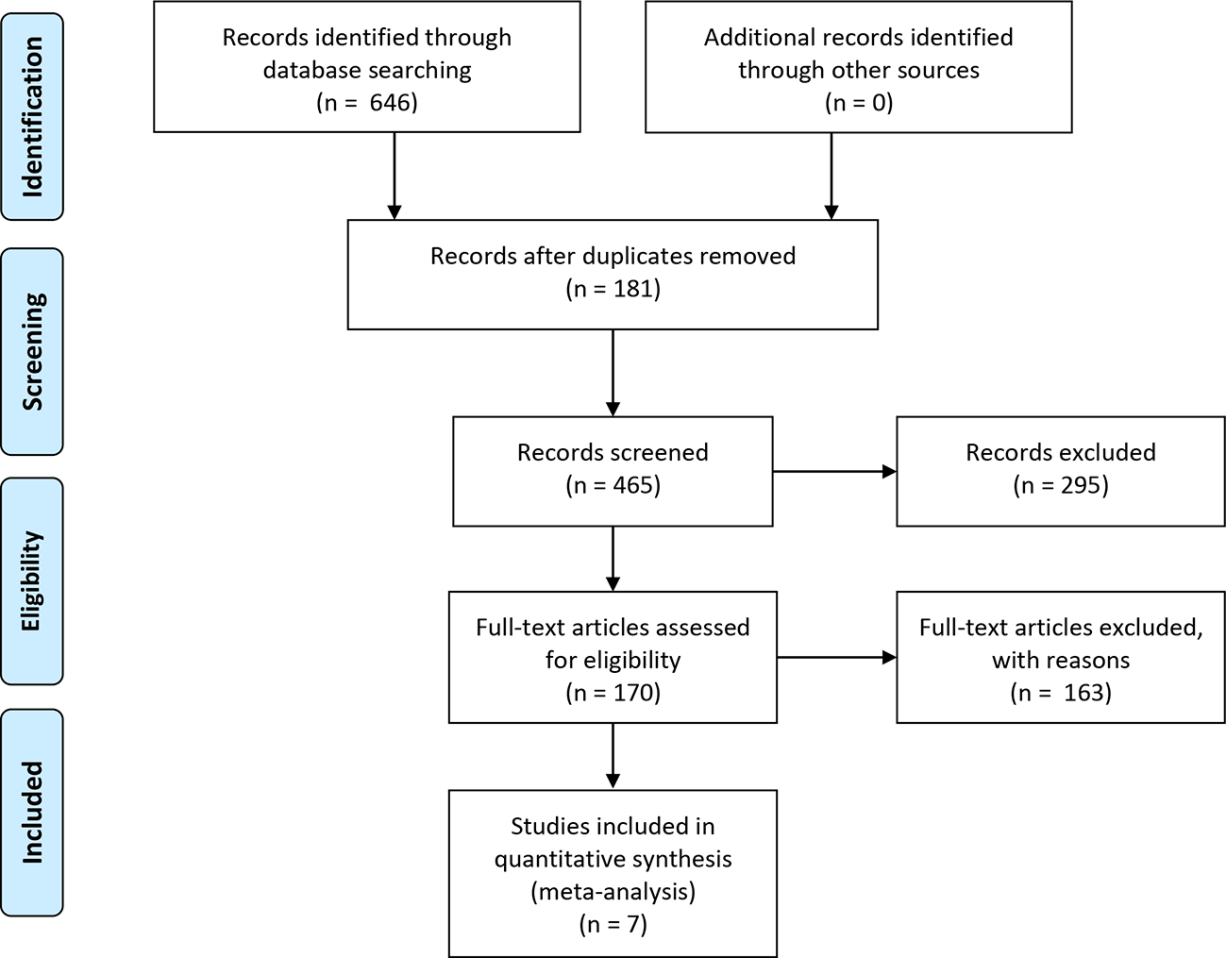
Flow chart of the literature search and study selection. The literature search and study selection procedure included four stages: literature identification through database searching based on the key terms, screening and study selection, eligibility confirmation, and enrollment of the final studies qualified for meta-analysis. In the identified 646 publications from database searching, 181 duplicates were removed and 465 records were further screened. As a result, 295 articles were excluded for reasons and the remaining 170 full-text articles were assessed for eligibility. Again, 163 studies were excluded for other reasons, and only seven studies including 3890 participants were eventually enrolled in quantitative synthesis.

The literature search resulted in the identification of 646 publications (**Figure 1**), from which 181 duplicates were removed and 295 articles were excluded as they were either animal experiments (70), abstracts with unavailable indicators (34), reviews (51), or topics not pertinent to the research question (140). After 170 full-text articles were screened, seven studies including 3890 participants were enrolled in this meta-analysis. All the articles were published in English, between 2013 and 2016. **Table 1** shows detailed characteristics of the clinical trials included.

**Table 1:** Characteristics of studies included in the meta-analysis.

Reference	Pain condition	Sample size	Age, years	Female ratio (%)	Mean duration of OA or LBP, years	Intervention	Outcomes
Kivitz et al., 2013	Chronic LBP	1052	51.7	52.9	10.9-12.3	P, T 5 mg iv q8w, T 10 mg iv q8w, T 20 mg iv q8w, N 500 mg bid	W-P, W-PF, PGA
Spierings et al., 2013	OA	610	57.4	62.6	6.2-7.6	P, T 5 mg iv q8w, T 10 mg iv q8w, O-CR 10-40 mg q12h	W-P, W-PF, PGA
Balanescu et al., 2014	OA	604	62.4	77.7	6.1-6.7	P+DSR^a^, T 2.5 mg+DSR, T 5 mg+DSR, T 10 mg+DSR	W-P, W-PF, PGA
Ekman et al., 2014	OA	828	61.1 ± 10.1^b^	60.3	7.2-9.0	P, T 5 mg iv q8w, T 10 mg iv q8w, N 500 mg bid	W-P, W-PF, PGA
Tiseo et al., 2014	OA	215	59.3 ± 8.7	68.8	NA^c^	P, F 0.03 mg/kg, F 0.1 mg/kg, F 0.3 mg/kg	W-P, W-PF,
Mayorga et al., 2016	OA	196	59.4 ± 9.2	56.3	NA	P, F 3 mg q4w, F 9 mg q4w, O-CR bid	W-P, W-PF, PGA
Sanga et al., 2016	OA	385	53.1 ± 12.0	54.0	NA	P, F 1mg q4w, F 3 mg q4w, F 6 mg q4w, F 10 mg q4w	PGA

LBP, low-back pain; OA, Osteoarthritis; W-P, WOMAC Pain; W-PF, WOMAC Physical Function; PGA, Patient’s Global Assessment; P, Placebo; T, Tanezumab; N, Naproxen; O-CR, Oxycodone-CR; F, Fasinumab; ^a^DSR, diclofenac sustained release; ^b^mean ± standard deviation; ^c^NA, not available.

### Risk of Bias of Individual Studies

**Figure 2:**
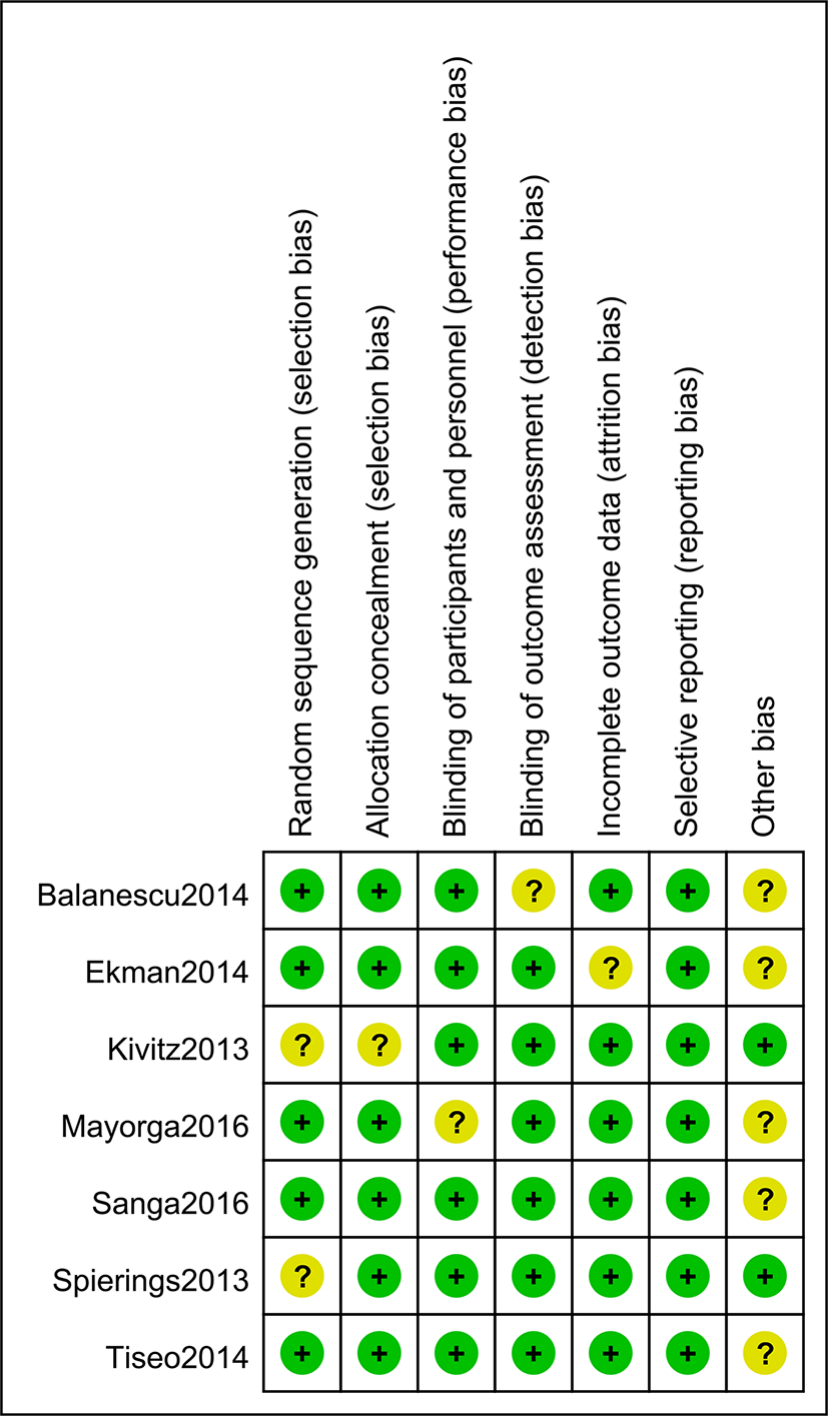
Risk of bias of individual studies. Five of the seven studies included were evaluated as “low risk” in selection bias (random sequence generation), and only one study showed an unclear risk of bias in allocation concealment. Six of the seven studies showed a low risk of performance bias, and only one study manifested an unclear risk in detection bias assessment. Six studies showed low risk in attrition bias and all the studies showed low risk of reporting bias.

**Figure 3:**
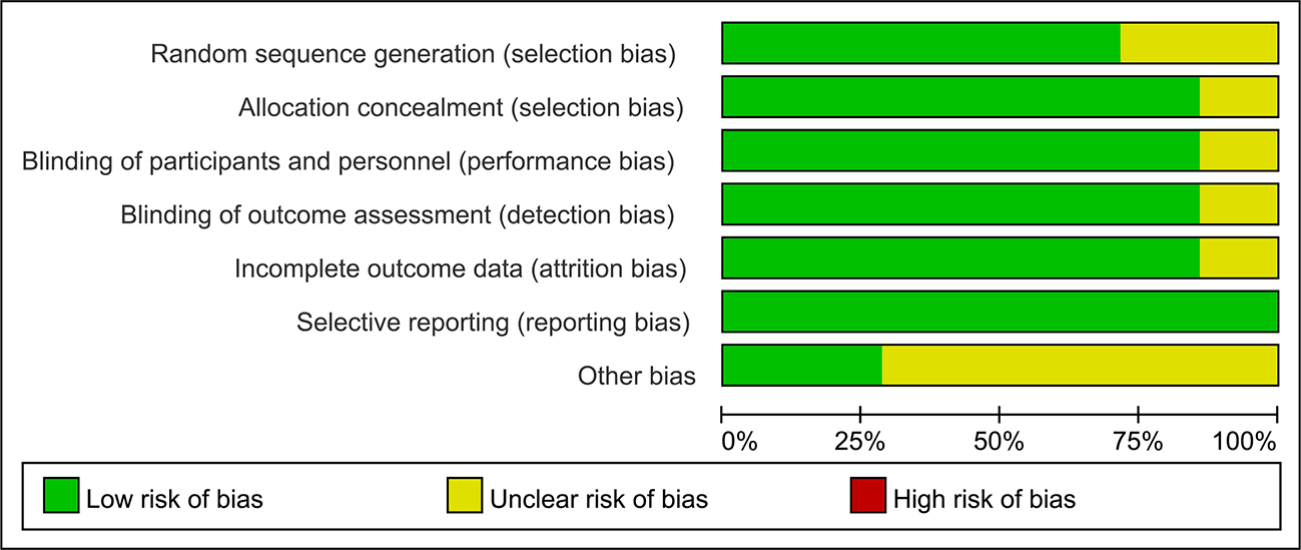
Risk of bias summarized in percentage. 71% (5/7) of the studies included were evaluated with low risk in selection bias (random sequence generation) and 85.7% (6/7) of studies showed low risk for allocation concealment. 85.7% of studies showed low risk of performance bias and detection bias. 85.7% of studies were assessed with low risk in attrition bias and 100% the studies showed low risk of reporting bias.

71% (5/7) of the studies included were evaluated with low risk in selection bias (random sequence generation), and only one study showed an unclear risk of bias in allocation concealment. Six of the seven studies showed low risk of performance bias. In regard to detection bias, only one study manifested an unclear risk in blinding of the outcome assessment. Six studies were assessed with low risk in attrition bias, and all the studies showed low risk of reporting bias. The risk of bias assessments is detailed in **Figures 2** and **3**.

### Assessment of Overall Effect Sizes

**Figure 4:**
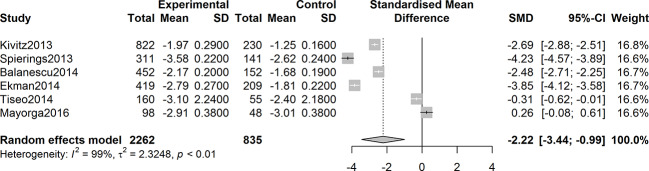
Forest plot of changes from baseline to checkpoint in the WOMAC Pain subscale. Forest plot showing the pooled effects on mean alterations in the WOMAC pain Index. Six studies were assessed and the result showed a significant reduction in the WOMAC pain Index (I^2^ = 99%, P = 0.0004).

Six studies were assessed for the efficacy of anti-NGF agents on the change in the WOMAC pain Index. The result showed a significant reduction in the WOMAC pain Index (SMD = -2.22, 95% CI = -3.44 to -0.99, Z = -3.55, P = 0.0004; I^2^ = 99%). Six studies were tested for the effect of anti-NGF agents on the change in the WOMAC Physical Function Index. The result demonstrated a significant decrease (SMD = -2.76, 95% CI = -4.22 to -1.30, Z = -3.71, P = 0.0002; I^2^ = 99%). Furthermore, six studies detected the efficacy of anti-NGF agents on the change in the PGA Index. The result also showed a significant decrease in the PGA Index (SMD = -2.76, 95% CI = -4.42 to -1.09, Z = -3.24, P = 0.0012; I^2^ = 99%). The pooled effects on mean alterations in the WOMAC pain, the WOMAC physical function, and PGA Index are shown in **Figures 4** **–** **6**.

**Figure 5:**
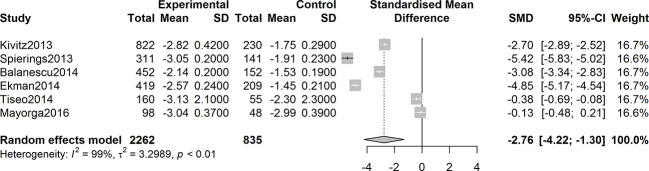
Forest plot of changes from baseline to checkpoint in the WOMAC Physical Function subscale. Forest plot showing the pooled effects on mean alterations in the WOMAC physical function subscale. Six studies were detected, and the result showed a significant reduction in the WOMAC physical function Index (I^2^ = 99%, P = 0.0002).

**Figure 6:**
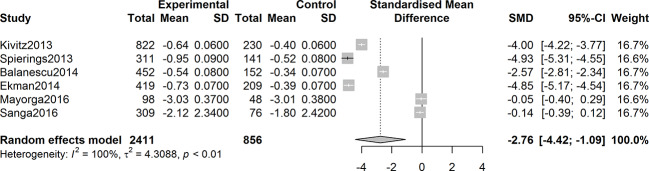
Forest plot of changes from baseline to checkpoint in the Patient’s Global Assessment Index. Forest plot showing the pooled effects on mean alterations in the Patient’s Global Assessment (PGA) Index. Six studies were tested and the result showed a significant reduction in the PGA Index (I^2^ = 100%, P = 0.0012).

### Adverse Events Incidence

**Figure 7:**
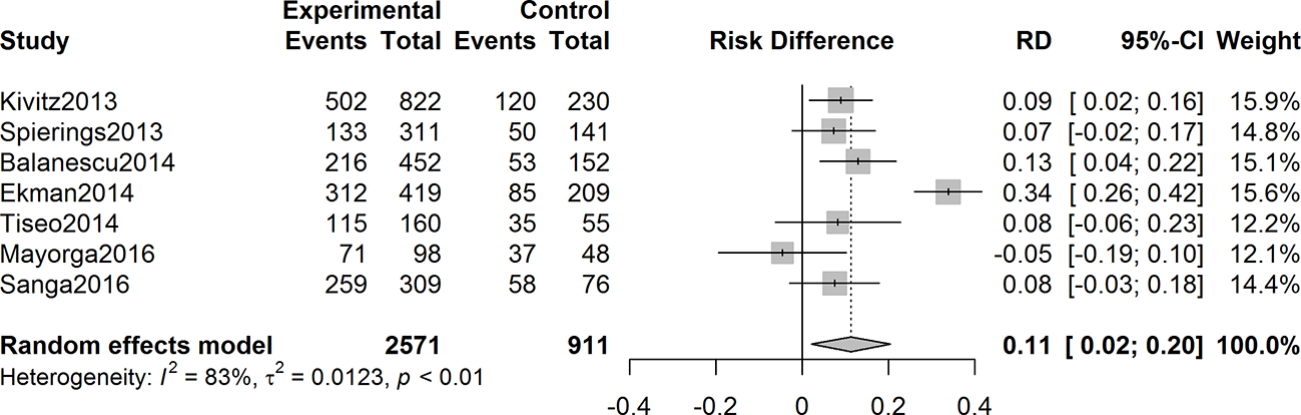
Forest plot of differences of adverse events rates in experimental and control groups. In the seven studies included, the overall incidence rate of adverse events was higher in the anti-NGF treatment group than that of the control group, with a pooled rate difference of 0.11 (95% CI: 0.02 to 0.20) (I^2^ = 83%, P = 0.016).

The overall incidence of patients with adverse events was higher in the anti-NGF agents’ therapy group than that in the control with a pooled rate difference of 0.11 (95% CI = 0.02 to 0.20, Z = 2.41, P = 0.016; I^2^ = 83%) (**Figure 7**). Nausea, paresthesia, arthralgia, arthralgia, and headache were the five most frequently reported adverse events in anti-NGF treatment group among the seven studies included.

### Sensitivity Analysis and Publication Bias

**Figure 8:**
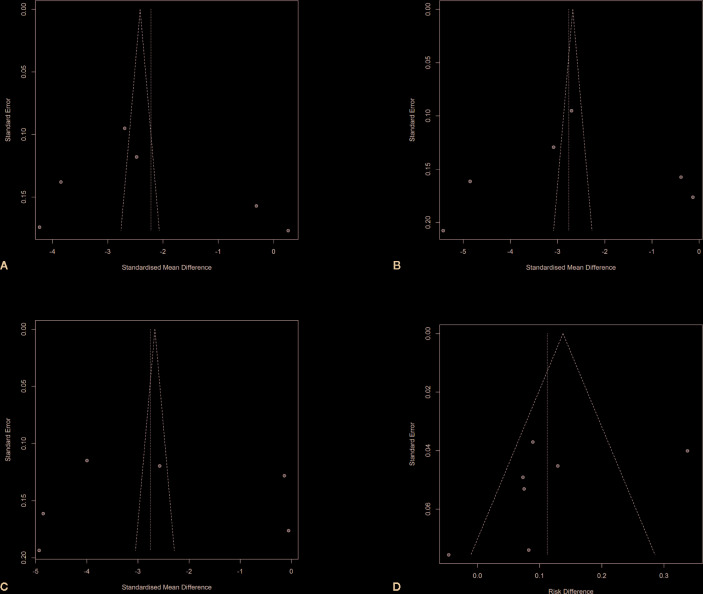
Funnel plots with pseudo 95% confidence limits. **(A)** Funnel plot of changes from baseline to checkpoint in the WOMAC Pain subscale. **(B)** Funnel plot of changes from baseline to checkpoint in the WOMAC Physical Function subscale. **(C)** Funnel plot of changes from baseline to checkpoint in the Patient’s Global Assessment Index. **(D)** Funnel plot of differences of adverse events rates in the anti-NGF agent treatment group and control group.

A sensitivity analysis was performed to investigate the impacts of single studies on the overall outcomes. After omitting each single study one after another, the pooled effects were not altered. The Rosenberg’s fail-safe numbers were 8399, 12493, 13205, and 116 for WOMAC pain, physical function, PGA Index, and adverse events evaluation, which indicated that large numbers of unpublished and non-significant studies would be added to for the P value of the effect to attain >0.05. Funnel plots were shown in **Figure 8**.

## Discussion

Meta-analysis is considered to be a powerful and widely-used tool by pooling results from different studies with specific statistical methods to draw conclusions which can be more meaningful than individual reports alone (Roberts et al., 2016; Lin and Chu, 2018). In this meta-analysis, seven published RCTs including 3890 participants with diagnosed OA pain and chronic LBP treated with anti-NGF antibodies were enrolled. The pooled results showed a significant reduction in the change of the WOMAC pain Index (P = 0.0004), WOMAC Physical Function Index (P = 0.0002), and the PGA Index (P = 0.0012). The results were consistent with preceding RCTs, which demonstrated that anti-NGF agents had a sounding effect on the relief of pain and function improvement in patients suffering from OA pain and chronic LBP. The underlying rationale may be that NGF plays a key role in the process of pain generation in chronic pain conditions (Amaya et al., 2004; Deandrea et al., 2008; Delaney et al., 2008) and anti-NGF antibodies have already revealed the potential to normalize nociceptive hyperactivity and produce pain alleviation in clinical settings, suggesting that these agents may play a role in pain treatment (Jimenez-Andrade et al., 2010; Katz et al., 2011).

In this meta-analysis, two authors independently did a systematical database search in both the Chinese and English language to increase the potential of retrieving all relevant studies. Data extraction was also conducted by two independent investigators using a designed form. A Cochran Q test and an I² statistic were used to explore the magnitude of the heterogeneity. The results indicated considerable levels of heterogeneity that could not be ignored. We conducted a random effects model to perform the pooled analysis. The potential elements contributing to heterogeneity may probably include differences in the sample sizes of RCTs, the demographic features of the study participants, the locations of studies, the duration of disease, the specific types and dosage of medications, ways of giving drugs, and other relevant factors. We originally tried to explore the potential impact of moderator variables using meta regression in the study design. Nevertheless, the information we retrieved was not complete for this process. Sensitivity analysis revealed that the pooled results demonstrated the robustness of the outcomes in this meta-analysis. Funnel plots showed some asymmetry, which indicates there may be publication bias. However, the results of Rosenberg’s fail-safe numbers calculation demonstrated that the publication biases (if they exist) may be theoretically ignored (Rosenberg, 2005; Muller et al., 2019). We were not able to extract data from several articles with incomplete parameters, which may influence our conclusions.

In comparison to previous meta-analysis or RCTs investigating the effect of a certain anti-NGF antibody on pain relief, our current analysis focused on the 3 types of anti-NGF antibodies and included patients with OA pain and chronic LBP. The overall effects were in consistency with each individual study. The pooled incidence of adverse events was higher in the anti-NGF agents’ treatment group than that in the control. The conclusion of this study may provide up-to-date evidence on chronic pain treatment for researchers and clinical practitioners in their fields of practice. Further clinical trials on anti-NGF antibodies will be continuously in need, and investigators should ensure rigorous methodology as well as controls for nonspecific therapy and therapist effects in order to reduce the risk of potential bias.

## Data Availability Statement

The original contributions presented in the study are included in the article/supplementary materials, further inquiries can be directed to the corresponding author.

## Author contributions

SY and YH conceived and designed this study. SY and LL were responsible for the collection, extraction, and analysis of the data. SY was responsible for writing the paper. ZY and YZ performed the quality evaluation and completed data analysis. YH polished the English language. All authors contributed to the article and approved the submitted version.

## Funding

This study was supported by the National Natural Science Foundation of China (No. 81803501), Natural Science Foundation of Zhejiang Province, China (No. LQ18H310001), Hospital Pharmacy Foundation of Zhejiang Pharmaceutical Association (No. 2019ZYY15), and Wu Jieping Medical Foundation (No. 320.6750.19090-23). We would like to thank all the researchers and study participants for their contributions.

## Conflict of Interest

The authors declare that the research was conducted in the absence of any commercial or financial relationships that could be construed as a potential conflict of interest.

## Abbreviations

CIs, confidence intervals; CNKI, China National Knowledge Infrastructure; DSR, diclofenac sustained release; LBP, low-back pain; NGF, nerve growth factor; OA, osteoarthritis; PRISMA, Preferred Reporting Items for Systematic Reviews and Meta-analysis; RCTs, randomized controlled trials; RD, rate difference; SMD, standardized mean difference; WOMAC, Western Ontario and McMaster Universities Arthritis Index.
